# Action-Sentence Compatibility: The Role of Action Effects and Timing

**DOI:** 10.3389/fpsyg.2013.00272

**Published:** 2013-05-21

**Authors:** Christiane Diefenbach, Martina Rieger, Cristina Massen, Wolfgang Prinz

**Affiliations:** ^1^Department of Psychology, Max Planck Institute for Human Cognitive and Brain SciencesLeipzig, Germany; ^2^Graduate Program: Function of Attention in Cognition, University of LeipzigLeipzig, Germany; ^3^Department for Medical Sciences and Management, Institute for Psychology, University for Health Sciences, Medical Informatics and TechnologyHall in Tirol, Austria; ^4^Leibniz Research Centre for Working Environment and Human FactorsDortmund, Germany

**Keywords:** action-sentence compatibility, language comprehension, motor simulation, action simulation, embodiment

## Abstract

Research on embodied approaches to language comprehension suggests that we understand linguistic descriptions of actions by mentally simulating these actions. Evidence is provided by the action-sentence compatibility effect (ACE) which shows that sensibility judgments for sentences are faster when the direction of the described action matches the response direction. In two experiments, we investigated whether the ACE relies on actions or on intended action effects. Participants gave sensibility judgments of auditorily presented sentences by producing an action effect on a screen at a location near the body or far from the body. These action effects were achieved by pressing a response button that was located in either the same spatial direction as the action effect, or in the opposite direction. We used a go/no-go task in which the direction of the to-be-produced action effect was either cued at the onset of each sentence (Experiment 1) or at different points in time before and after sentence onset (Experiment 2). Overall, results showed a relationship between the direction of the described action and the direction of the action effect. Furthermore, Experiment 2 indicated that depending on the timing between cue presentation and sentence onset, participants responded either faster when the direction of the described action matched the direction of the action effect (positive ACE), or slower (negative ACE). These results provide evidence that the comprehension of action sentences involves the activation of representations of action effects. Concurrently activated representations in sentence comprehension and action planning can lead to both priming and interference, which is discussed in the context of the theory of event coding.

## Introduction

### Embodied language comprehension

Imagine that a friend who plays football tells you that she has scored a goal. While listening to her report, you vicariously experience the described events. You “see” the shot in your mind’s eye, and if you have your own experiences with playing football, you probably “feel” the movement of kicking the ball. We are all familiar with this kind of vicarious experience of a described situation not only from conversations, but also from reading stories when we feel as if the events occurring in the story happened to ourselves.

This kind of vicarious experience is what the proponents of embodied approaches to language comprehension (Barsalou, 1999; Glenberg and Kaschak, 2002; Zwaan, 2004; Pulvermüller, 2005) call *mental simulation* of the described situation, and it is regarded as essential for capturing the meaning of an utterance. According to the embodied view, words and sentences reactivate memory traces from actual experiences with the denoted objects, events, or actions in the person trying to comprehend the words or sentences. These perceptual and action representations enter into a mental simulation that is constructed during language comprehension. Empirical evidence for those assumptions stems particularly from studies on action-related language. Here it is assumed that the comprehension of action-related language relies on action simulation, that is, on the reactivation of stored motor experiences.

An embodied approach to language comprehension may have important implications for the role of conscious awareness in action processing. This is because an approach like this challenges the classical distinction between explicit, declarative knowledge about action (as is involved in representations of action-related words and sentences) and implicit, procedural knowledge for action (as is involved in motor representations for action control). Challenging this distinction is, to some extent, tantamount to challenging that there is a functional separation between conscious and non-conscious modes of action processing. Common opinion holds that processing of declarative knowledge is (mandatorily) conscious whereas processing of procedural knowledge does not require conscious awareness (Squire, 1992; Balota et al., 2000; Tulving, 2000; Baddeley, 2002). If so, the claim that one is grounded in the other seems to imply that conscious and non-conscious processing modes draw on common representational resources and are, in functional terms, less different than is often claimed and believed.

### Evidence for motor simulations in language comprehension

Neurophysiological and brain imaging studies have indicated that motor system activation is involved in semantic access to the meaning of action words. For instance, comprehension of sentences describing actions performed with the mouth, hand, or leg engages motor circuits that largely overlap with those activated during execution and observation of the described actions (Tettamanti et al., 2005). Further, changes in motor excitability are specific for the effector involved in the described action (Buccino et al., 2005). When the arm’s motor area is stimulated, words referring to arm actions are recognized faster than words referring to leg actions, and the opposite pattern occurs when the leg’s motor area is stimulated (Pulvermüller et al., 2005). In addition, processing of action verbs at the onset of reaching movements affects the kinematics of the movements 160–180 ms after word onset (Boulenger et al., 2006). At this time, early lexico-semantic processes are known to occur (Sereno et al., 1998). Even when action verbs are only displayed subliminally while participants prepare a reaching movement, they affect motor preparation and subsequent movement kinematics (Boulenger et al., 2008).

On the behavioral level, studies show content-specific interactions between the understanding of a verbally described action and a concurrently performed motor response. Usually these interactions reflect a facilitated execution of the motor response when the response shares some features with the semantic meaning of the action-related words and sentences presented as stimuli. An example of such an interaction is the action-sentence compatibility effect (ACE; Glenberg and Kaschak, 2002), which refers to compatibility between the direction of a described action and the direction of the response. In ACE experiments, participants judge whether sentences describing actions toward or away from the body, such as “Courtney handed you the notebook” or “You handed Courtney the notebook,” are sensible or not. Participants perform the judgment by moving the hand from a home button in the center of a response device to either a button closer to their body (near button, movement toward the body) or to a button further away from the body (far button, movement away from the body). Several studies have shown that when the movement direction for the response is compatible with the movement direction expressed in the sentence, e.g., when both are directed away from the body, response times are faster than when movement directions are incompatible (Glenberg and Kaschak, 2002; Borreggine and Kaschak, 2006; Glenberg et al., 2008b; Kaschak and Borreggine, 2008). A similar compatibility effect has been observed for verbally described directions of manual rotations (e.g., opening a water bottle) and rotation directions that were produced by turning a knob (Zwaan and Taylor, 2006). Zwaan et al. (2012) found that the response execution was modulated even when there was no feature overlap between responses and verbally described movements. Participants were presented with sentences that implied a forward or backward movement and although response movements involved leaning to the left or right on a balance board, these movements were shifted forward or backward depending on the sentence content.

### The nature of the involved action representations

Based on those and similar behavioral and neurophysiological studies, embodied theories of language comprehension, such as the theory of perceptual symbol systems (Barsalou, 1999), or the indexical hypothesis (Glenberg and Robertson, 1999, 2000), assume that linguistic contents evoke multimodal representations of their referents. In their view, language reactivates experiences that were encoded and stored by different modality-specific systems. In the case of action words or sentences, the motor system partially reactivates the motor state that produces the denoted action, thereby creating a simulation of that action (Barsalou, 2003). Thus, action representations activated during language comprehension are supposed to refer to specific motor programs.

In spite of the considerable body of evidence for the involvement of motor programs in understanding action-related language, it is conceivable that another kind of action representation is also involved. Based on assumptions of the common coding approach (Prinz, 1990, 1997), representations of described actions could be coded in terms of action goals or action effects. Prinz’s approach proposes that perceived events and planned actions are coded in a common representational format. This common coding of perception and action is thought to result from actions being represented in terms of their perceptual consequences or intended action effects (Prinz, 1990; for evidence see, e.g., Elsner and Hommel, 2001; Rieger, 2007). Support for this assumption comes from stimulus-effect compatibility effects. For instance, Hommel (1993) demonstrated that response times are faster when participants respond to a stimulus which has a spatial compatibility to an intended action effect (e.g., both are on the left side), regardless of whether the intended action effect was produced by a spatially compatible or non-compatible action (i.e., by pressing a right or left key).

Some studies have already indicated that interactions between language processing and actions can occur on the level of intended action effects. Markman and Brendl (2005) found that responses to positive words were facilitated when producing an action effect with a positive connotation (approaching the participant’s name on the screen) compared to producing an action effect with a negative connotation (withdrawing from the participant’s name, i.e., avoidance action). This compatibility effect was independent of whether the action effect resulted from moving the arm toward or away from the body. In this case, priming occurred because representations of emotional words and response representations shared affective codes on the level of action effects (see also Eder and Klauer, 2007, 2009; Eder and Rothermund, 2008; van Dantzig et al., 2008). Lindemann et al. (2006) showed that semantic processing of words was facilitated when the words denoted the goal of an action that was prepared before. Thus, the activated action goal primed the word meaning, which again suggests common codes that represent the intended action effects.

### The present study

The experiments investigating compatibility effects between language comprehension and concurrent action, so far, either have not clearly differentiated between the representations of actions and intended action effects or they only used affective word stimuli (like Markman and Brendl, 2005). Therefore, it is unclear to what extent the comprehension of sentences is based on representations of intended action effects or on motor representations.

Our experiments addressed this question by testing whether action representations activated during sentence comprehension interact with representations of intended effects of actions or with representations of the motor component of these actions. We asked participants to indicate whether sentences describing actions toward or away from the body were sensible or not. The sentences expressed transfer of concrete or abstract objects between the participant and another person. Participants were asked to perform the judgment by producing an action effect (lighting a star on a horizontally mounted screen) at a location either near the body or far from the body. These action effects were achieved by moving the hand from a centrally located button to a button located nearer to or further away from the body. In order to dissociate actions from their intended effects, participants performed the task with either a regular spatial relationship between actions and the intended effects (e.g., combining a movement to the near button with an action effect located near the body) or with an inverted spatial relationship between actions and effects (e.g., combining a movement to the near button with an action effect located far from the body).

We looked at sentence-effect compatibility, that is, the compatibility between the direction of the action described in the sentence (object transfer toward or away from the body) and the direction of the intended action effect (a star appearing on the screen at a location near the body or far from the body). If representations of intended action effects play a role in understanding action-related language, responses should be faster in the sentence-effect compatible condition than in the sentence-effect incompatible condition. This pattern of results (an action effect-related ACE) should be observed both in regular and in inverted conditions. If, however, representations of the motor component of actions predominantly contribute to the understanding of action-related language, compatibility should be effective between the sentence direction and the direction of the arm movement to the response button (movement-related ACE). In this case, different patterns should be observed in the regular and inverted condition: in the regular action-effect relation condition in which the directions of actions and action effects are completely correlated, responses should be faster in the sentence-effect compatible condition than in the sentence-effect incompatible condition. This pattern should reverse in the inverted action-effect relation condition. Because sentence-effect compatibility is equivalent to sentence-action incompatibility in the inverted condition, the reversed ACE pattern means that responses are faster with sentence-action compatibility than sentence-action incompatibility.

The question of whether the ACE is related to the arm movement or to the intended action effect might be answered differently for concrete and abstract sentences. Understanding concrete sentences might involve activation of specific motor programs and hence give rise to a movement-related ACE, whereas understanding abstract sentences might involve activation of representations of action effects and therefore lead to an action effect-related ACE.

## Experiment 1

Experiment 1 investigated whether the comprehension of action sentences relies on actions or on intended action effects by dissociating actions from their intended effects in an ACE paradigm. We wanted participants to be aware of the locations of the to-be-produced action effect and avoid participants adapting to certain movements when judging sentences. Therefore, we varied randomly whether the response required producing an action effect at the near or the far location. In order to keep the task as easy as possible, we adopted the go/no-go method from Borreggine and Kaschak’s (2006) ACE experiments, in which participants only responded when they judged sentences to be sensible. Participants were informed about the current location of the to-be-produced action effect by a visual cue at the onset of every sentence, similar to a condition in which Borreggine and Kaschak found the standard ACE.

### Method

#### Participants

Nineteen adults were paid 7 Euros to participate in the experiment. The data from three participants were excluded from analyses for reasons explained later in the data analysis section. Thus, the final sample comprised 16 participants (mean age = 24.8 years; 6 males, 10 females). All participants were native German speakers, right-handed, and had normal or corrected-to-normal vision and audition. They were randomly assigned to two groups containing eight participants each. One group performed the task in the regular action-effect relation condition, while the second group performed the task in the inverted action-effect relation condition.

#### Stimuli and apparatus

The linguistic material comprised 80 triads of sentences that were adopted from Glenberg et al. (2008b) and translated into German. Each triad consisted of three versions of an action sentence: the two critical sentences of each triad described the same transfer action directed either toward the body (e.g., “Jakob reicht dir das Buch” [Jacob hands you the book]), or away from the body (e.g., “Du reichst Jakob das Buch” [You hand Jacob the book]). The third sentence contained the same character names and objects as the transfer sentences, but a different verb that expressed no transfer (e.g., “Du liest mit Jakob das Buch” [You read the book with Jacob]). Half of these neutral sentences began with the German word “Du” [you], like the away sentences, and half began with a character name, like the toward sentences. In addition, half of the triads described the concrete transfer of objects (as in the examples above), and half described abstract transfer, for example, the transfer of information (e.g., “Julia erzählt dir eine Geschichte” [Julia tells you the story]). Half the sentences (40 triads) were sensible and half were nonsensical. Twenty additional sentences were created and served as practice items. All sentences were recorded by a female German speaker and presented over headphones during the experiment.

**Figure 1:**
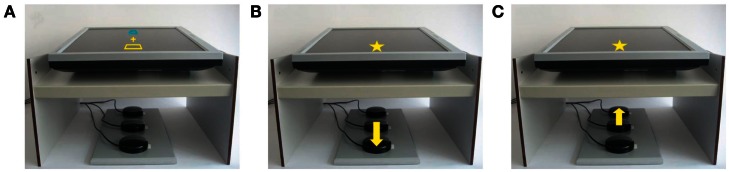
**Illustration of response fields and cue (A) and of arm movement and its effect by the example of a “yes” response in the yes-is-near condition with regular action-effect relation (B) and with inverted action-effect relation (C)**.

The response device (see Figure 1) consisted of three buttons (diameter: 6.3 cm) that were arranged in a vertical line on a board. The distance between the center of the middle button and the centers of the near and far button was 11.3 cm. The board was located on a table in front of the participant so that the buttons differed in distance from the participant’s body. The near button was about 20 cm away from the body. Above the buttons, a 17″ flat screen monitor was mounted horizontally. On the screen, two response fields were presented on a black background. One of the fields appeared at a near location (subtending a visual angle of 6.6°) and one at a far location (visual angle of 3.1°). The distance between the centers of the response fields was identical to the distance between the centers of the outer response buttons (i.e., 22.6 cm). One response field had a blue frame and the other one was framed in yellow; both fields were black inside. To indicate that a sentence was sensible participants were asked to activate a given response field by pressing the near or far button. The color (blue or yellow) of a cross (1.8° of visual angle) served as a cue to indicate the response field that had to be activated. As an effect of the button press, a star flashed in place of the activated response field on the screen. The effect star had the same color as the activated response field and subtended a visual angle of 16.2° (near location) or 7.7° (far location). To increase attention to the visual response effect, a sound (“twinkles”) was presented at the same time as the star appeared. The sound was composed of two successively presented tones that formed a fourth upward (with fundamental frequencies of 625 and 834 Hz).

Because the screen was placed above the buttons, the moving hand was covered and, thus, participants received no on-line visual feedback of their movement, but only perceived its effect on the screen. The experiment was controlled by an IBM-compatible computer running Presentation software (Neurobehavioral Systems, Albany, USA), and the response buttons were connected to it via the parallel port.

#### Procedure and design

**Figure 2:**
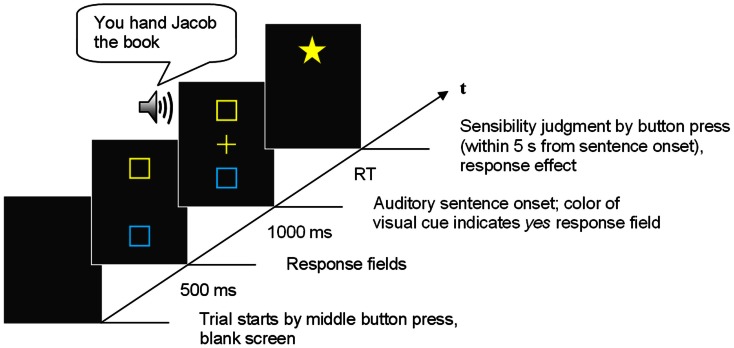
**Sequence of events in an experimental trial of Experiment 1**. This trial gives an example of a sensible away sentence presented in the yes-is-far condition.

The experiment was run in a dimly illuminated and sound-attenuated room. Each trial was initiated by pressing the middle button with the right hand, and participants were told not to release this button until they were able to make their response. Five-hundred milliseconds after the button press, the blue and the yellow response fields appeared on the screen at the near and far location and 1000 ms after their appearance, the auditory presentation of a sentence started. Participants were instructed to decide if the presented sentence was sensible or not. As a go/no-go task was used, participants were asked to respond only when the sentence was sensible (*yes* response), and to refrain from responding to a nonsense sentence. The yes response was randomly assigned to either the near response field (yes-is-near condition) or to the far response field (yes-is-far condition). When the sentence presentation started, the response cue (a cross) appeared in the center of the screen matching the color of one of the response fields. The color of the cue indicated whether the near or the far response field should be activated if the sentence was sensible. Activating the response fields required moving one’s arm from the middle button to the near or far button, that is, toward the body or away from the body. Participants were asked to give the yes response as soon as the sensibility of the sentence could be decided, thereby responding as quickly and accurately as possible. In case a response occurred, a star flashed and the accompanying sound was presented as soon as one of the response buttons was pressed. The star replaced the response field on the screen. The cue remained visible until the response was made or, in the case of a sentence being judged as nonsensical and no response being given, until the trial timed out 5 s after sentence onset. The sequence of trial events is illustrated in Figure 2.

There were two different mappings of action effects to buttons (see Figure 1 for an illustration): in the condition with the regular action-effect relation, actions and action effects were completely correlated, which means that the location of the to-be-produced action effect on the screen corresponded with the location of the button press (i.e., both were near the body or both were far from the body). In contrast, the action and its effect were opposed in the condition with the inverted action-effect relation: an action effect at a certain location on the screen resulted from moving one’s arm in the opposite direction (i.e., the star appeared at the near location on the screen when pressing the far button and vice versa).

At the beginning of the experiment, participants received two blocks of practice trials. The first block consisted of 32 trials in which participants were familiarized with the response assignment. They were only presented with the German words “Ja” [yes] and “Nein” [no]. In the yes trials they were asked to activate the response field that was indicated by the visual response cue, in the no trials they were asked to refrain from responding. Feedback about the correctness of the response was provided by displaying the German word “Richtig” [right], colored green, or “Falsch” [wrong], colored red, on the screen. In the second practice block, participants received 20 trials with practice sentences. The two response assignments were each presented in one half of the trials. The whole experiment lasted approximately 30 min.

Apart from action-effect relation (regular, inverted), which was manipulated between participants, all of the independent variables were manipulated within participants. Sentence direction (toward, away, and neutral), sentence type (concrete and abstract), sensibility (sensible and nonsensical), and effect direction (yes-is-near, yes-is-far) varied from trial to trial.

To ensure that all sentences appeared equally often in every condition, the 240 stimulus sentences were divided into two material blocks which were assigned to one of the effect directions each. In each trial, a sentence was selected randomly from one of the two material blocks. The assignment of material blocks to conditions of effect direction and action-effect relation was counterbalanced across participants. Sentences were randomized in such a way that each material block was divided into five subblocks (24 sentences each) that contained an equal number of sentences of each category (sensibility, sentence type, sentence direction), but never included sentences that belonged to the same triad. For each participant, the order of sentences in each subblock, as well as the order of the subblocks themselves, was randomized.

#### Data analysis

In both Experiments 1 and 2, participants were removed from the analysis and replaced by a new participant (a) when their error rates exceeded 15% or (b) when participants had their hand resting on the middle button and only pressed the response buttons with fingers splayed out in more than 15% of the trials, despite being instructed to move the whole hand from the middle button to the response button. These cases were identified through earlier registration of response button presses than the release of the middle button.

Dependent variables were total response time (TRT)^1^ and percentages of errors. TRT was measured from the onset of sentence presentation to the pressing of the near or far response button. Incorrect trials were excluded from the analysis. To reduce the effect of outliers, first, 0.5% of the longest and shortest responses over participants were eliminated, and second, for each participant in each condition, responses that deviated more than 2.5 SD from the condition mean were discarded. This procedure was based on the trimming procedure used by Glenberg et al. (2008b).

Only data from the sensible toward and away sentences were analyzed (see Glenberg et al., 2008b). In order to simplify the analysis and to make the data more easily accessible, the variables sentence direction and effect direction were merged into a new variable, sentence-effect compatibility (compatible, incompatible). The sentence-effect compatible condition always contained cases in which effect direction matched the sentence direction, irrespective of the direction of the arm movement required for the response. The sentence-effect incompatible condition included cases in which effect direction and sentence direction were opposed.

Three-way mixed-factor analyses of variance (ANOVAs) were conducted on TRTs and error rates with sentence-effect compatibility (compatible, incompatible) and sentence type (concrete, abstract) as within-subjects factors and with action-effect relation (regular, inverted) as a between-subjects factor. Since compatibility effects are the main interest of this work, only main effects of and interactions with sentence-effect compatibility will be reported.

### Results

#### Total response time

**Figure 3:**
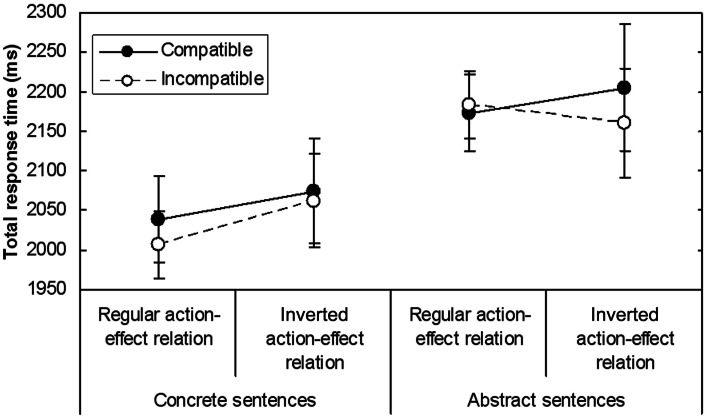
**Mean TRTs (in ms) in Experiment 1 as a function of the factors Sentence-effect compatibility, Action-effect relation, and Sentence type**. Error bars indicate standard errors.

The trimming procedure applied to the data from the final sample resulted in the elimination of 4.8% of the TRT data. A significant main effect of sentence-effect compatibility [*F*(1, 14) = 4.67, MSE = 1344.46, *p* = 0.049] and a significant interaction between sentence-effect compatibility, sentence type, and action-effect relation [*F*(1, 14) = 6.73, MSE = 843.77, *p* = 0.02] were found (see Figure 3 for mean TRTs). Further ANOVAs, performed separately for each sentence type, revealed that responses to concrete sentences were faster across action-effect relations in the sentence-effect incompatible condition (*M* = 2034, SD = 144) than in the sentence-effect compatible condition [*M* = 2056, SD = 167; *F*(1, 14) = 4.59, MSE = 851.22, *p* = 0.05]. This was not modified by an interaction between sentence-effect compatibility and action-effect relation [*F*(1, 14) = 0.92, MSE = 851.22]. In contrast, for abstract sentences a significant interaction between sentence-effect compatibility and action-effect relation was observed [*F*(1, 14) = 4.63, MSE = 1337.0, *p* = 0.049]. When the action-effect relation was inverted, responses were faster in the sentence-effect incompatible condition compared to the sentence-effect compatible condition [*t*(7) = 2.98, *p* = 0.02]. No significant difference was found in the regular action-effect relation condition [*t*(7) = −0.49]. In sum, TRTs for concrete sentences showed a sentence-effect compatibility disadvantage (negative ACE) that was not modulated by action-effect relation, and TRTs for abstract sentences displayed a sentence-effect compatibility disadvantage only in the condition with inverted action-effect relation.

#### Effects of response speed on the ACE

The observation of a negative ACE was surprising, particularly since we followed the procedure by Borreggine and Kaschak (2006) that yielded a positive ACE. However, Borreggine and Kaschak manipulated the timing of response planning in their ACE experiments. When the response cue that informed participants of the movement direction was presented at the onset of the sentence, the response could be planned while the sentence was processed. In this condition, a positive ACE arose. In contrast, when the cue appeared after the offset of the sentence, which prevented participants from preparing the response during sentence processing, responses were slower and the ACE was eliminated and descriptively showed a tendency to be reversed.

The negative ACE in the current experiment could result from participants not immediately paying attention to the response cue when it appeared on the screen. Since the cue was visible throughout the whole sentence presentation, there may have been a tendency for participants to postpone processing of the cue and response preparation to the end of the sentence. In this way, our experiment may have corresponded to Borreggine and Kaschak’s (2006) condition with delayed cue presentation, in which the ACE started to become negative. Thus, different timings between response preparation and sentence comprehension might be responsible for this result.

In order to investigate whether this might be the case, we used participants’ mean TRTs of all correct trials containing sensible toward and away sentences to obtain a measure reflecting how fast each participant responded on average. Fast responses may reflect relatively early response preparation, whereas slow responses may reflect relatively late response preparation. We repeated the ANOVA described above with participants’ speed as an additional covariate. The analysis revealed a significant interaction between sentence-effect compatibility and speed [*F*(1, 13) = 12.53, MSE = 737.24, *p* = 0.004]. To clarify the nature of this interaction, we correlated participants’ speed (i.e., their mean TRTs) with the magnitude of the ACE (difference between TRTs for sentence-effect incompatible trials and TRTs for sentence-effect compatible trials, i.e., positive numbers indicate a compatibility advantage and negative numbers a compatibility disadvantage). A negative correlation was obtained (*r* = −0.7, *p* = 0.003) which reveals that the slower the participants responded, the more they showed a compatibility disadvantage.

#### Error rates

In the ANOVA on error rates, no significant effects involving sentence-effect compatibility were found (all *F*s < 1.1). Mean error rates are given in Table 1.

**Table 1:** **Mean error rates (in %) and standard errors of error rates (in parentheses) in Experiment 1**.

	Concrete sentences		Abstract sentences	
	Compatible	Incompatible	Compatible	Incompatible
Regular action-effect relation	0.00 (0.00)	0.63 (0.63)	0.00 (0.00)	0.66 (0.66)
Inverted action-effect relation	0.00 (0.00)	0.63 (0.63)	0.63 (0.63)	0.00 (0.00)

### Discussion

The present experiment addressed the contributions of actions and intended action effects to the ACE. To this end, actions were dissociated from their effects and differed in whether they were directed toward the body or away from the body. In each trial, participants were instructed about the current direction of the yes response at sentence onset. The results were different for concrete and abstract sentences. TRTs for concrete sentences showed a negative ACE that referred to the action effect, because the data pattern was the same with the regular and inverted action-effect relations. Thus, the mental simulation during the comprehension of concrete sentences seems to involve representations of action effects. For abstract sentences, the ACE occurred only in the condition with inverted action-effect relation, but not in the condition with regular action-effect relation. Therefore, it cannot be determined whether the ACE in the inverted condition relied on action or effect, and no conclusions can be drawn regarding the nature of the representations activated during the comprehension of abstract sentences.

A follow-up analysis was conducted to examine whether the unexpected occurrence of the negative ACE was connected with response timing. The results suggest that slow responses, which probably reflect response preparation after the completion of the sentence, promote the emergence of a negative ACE. This indicates that the relative timing between movement preparation and sentence comprehension might play a role for the reversal of the ACE.

Changes in compatibility effects depending on relative timing have also been observed in other studies. For instance, Richardson et al., 2001, Experiment 1) presented participants with a series of pictured objects that afforded an action on either the left or the right side. Afterward, participants were asked to press a left or right key in order to indicate whether they had seen a certain object or not. Responses were facilitated when the side of the required keypress was opposite to the side of the action afforded by the recalled object. This incompatibility effect seemed to depend on the timing of the responses: when, in a second experiment, response time data were split into an early and a late half, the late group, again, exhibited an incompatibility effect between motor responses and affordances of verbally described objects. The early group, in contrast, displayed a non-significant tendency toward a compatibility effect.

To explain their results, Richardson et al. (2001) and Borreggine and Kaschak (2006) drew on the theory of event coding (TEC; Hommel et al., 2001). TEC suggests that action representations with overlapping features prime each other when they are activated at short time intervals (compatibility benefits), but interfere with each other when they are activated at long intervals (compatibility costs). In the light of TEC, compatibility benefits and costs in the ACE may arise as follows: during online sentence processing, feature codes are activated that represent the action that the sentence content is referring to. Among those feature codes is the directional code (toward or away from the body). In the first phase (activation phase), these codes can be activated more easily for planning another action that shares features with the first action. If the response is prepared during this activation phase, access to the activated directional feature code of the described action is easier. Thus, responding in the same direction is facilitated, resulting in compatibility benefits (the standard ACE). At the end of the sentence, when all relevant information is known, the activated feature codes are probably bound together to form a complete representation of the sentence content (which means running a full simulation of the described action). In this second phase (integration phase, about 250–500 ms after feature activation, Stoet and Hommel, 2002), the feature codes can no longer be activated in isolation. If response planning does not take place until the sentence is completed, the directional feature code is less available for coding the response. Thus, responding in the same direction is impaired, resulting in compatibility costs. This could account for the pattern of results obtained in Experiment 1: a large part of participants probably held off preparing their response until the end of the sentence, which caused the negative ACE.

## Experiment 2

Theory of event coding implies that there are mutual interactions between sentence comprehension and response planning. Therefore, not only should semantic processing of the sentence be able to facilitate or impair response planning, but also vice versa, depending on the temporal order of the two processes. In order to investigate the consequences of the timing between movement preparation and sentence comprehension for the ACE, the stimulus onset asynchrony (SOA) between the onset of sentence presentation and cue presentation was manipulated in Experiment 2. Moreover, the response cue did not remain on the screen but was presented only for a short period of time in order to limit the processing of the response cue to a certain point in time. In addition to addressing the timing issue, Experiment 2 continued to pursue the initial question of whether the ACE relies on actions or on action effects. Thus, again, the spatial relationship between action and action effect was manipulated.

### Method

#### Participants

Fifty German native speakers took part in the experiment in return for 7 Euros. The data from 10 participants were discarded for the reasons stated previously, and so analyses were based on the data from 40 participants (mean age = 24.4 years; 15 males, 25 females). All participants were right-handed and had normal or corrected-to-normal vision and audition. They were randomly assigned to the regular or the inverted action-effect relation condition, each comprising 20 participants.

#### Stimuli and apparatus

The stimuli and apparatus were identical to those used in Experiment 1.

#### Procedure and design

The procedure and design were the same as in Experiment 1, apart from the following modifications: the cue signaling the direction of the yes response in each trial was visible only for 500 ms. The SOA between sentence onset and the presentation of the response cue was manipulated within subjects and varied blockwise. In one of the five SOA conditions, the response cue appeared on the screen simultaneously with the onset of the sentence presentation (SOA = 0 ms; as in Experiment 1). In the other conditions, the cue was presented 1000 ms before sentence onset (SOA = −1000 ms), 500 ms before sentence onset (SOA = −500 ms), 500 ms after sentence onset (SOA = 500 ms), and at the end of the sentence presentation (SOA = 100% of the sentence length).

At the start of the experiment, participants received 40 trials to practice the response mode. They then performed 20 trials with practice sentences. The experimental design was identical to that of Experiment 1, apart from the additional independent variable SOA (−1000, −500, 0, 500 ms, 100% of sentence length). The order of SOA blocks was counterbalanced between participants. For each participant, an equal number of sentences of each category, in each material block, were pseudorandomly assigned to the SOA conditions in such a way that, across participants, each combination of effect directions, SOAs, and action-effect relations contained each sentence with equal frequency.

#### Data analysis

In the present experiment, median TRTs instead of mean TRTs were computed for each participant in each condition, because the additional SOA manipulation resulted in too few data points per condition to identify and remove outliers. Further, trials with two particular triads of sentences (one concrete and one abstract) were excluded from analyses. They were erroneously judged as nonsensical by a large proportion of participants, which led to unbalanced frequencies of these sentences in the different conditions. Since there were relatively few data points per condition in this experiment, some conditions did not include any correct response to these sentences at all, while other conditions did. This could have distorted the results due to the different sentence lengths. This resulted in the elimination of 5.0% of the data. Further data analysis was identical to that of Experiment 1, except that the performed ANOVAs included SOA as an additional within-subjects factor.

### Results

#### Total response time

**Figure 4:**
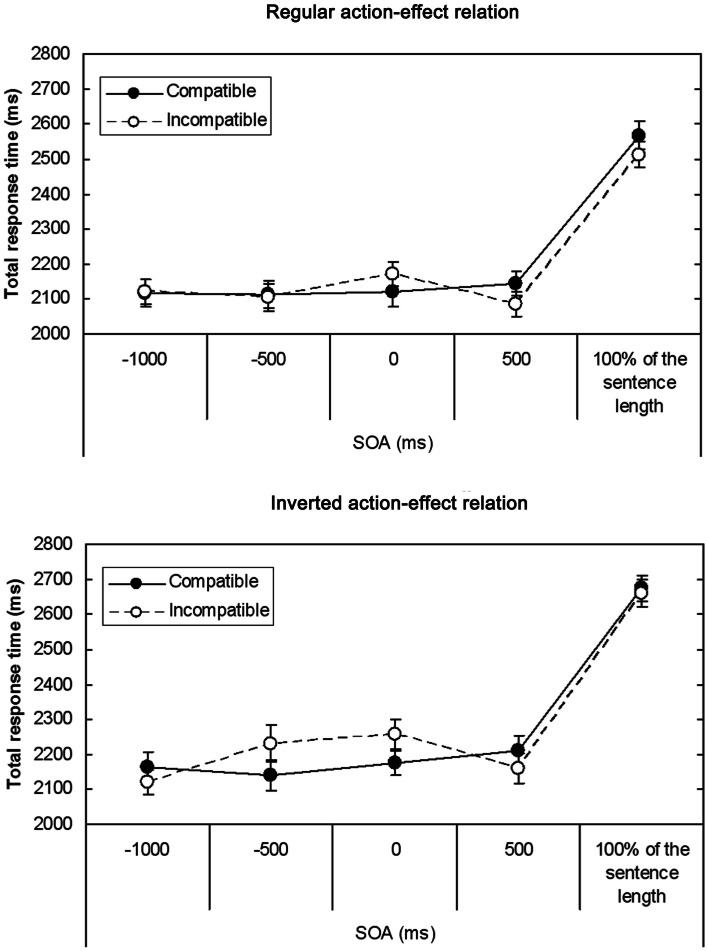
**Mean TRTs (in ms) in Experiment 2 as a function of Sentence-effect compatibility, SOA, and Action-effect relation (top panel: regular action-effect relation; bottom panel: inverted action-effect relation)**. Data are averaged over concrete and abstract sentences. Error bars represent standard errors.

Mean TRTs are depicted in Figure 4; since no effect of sentence type was observed in the ANOVA, data are presented averaged over concrete and abstract sentences. The ANOVA showed a significant interaction between sentence-effect compatibility and SOA [*F*(4, 152) = 4.58, MSE = 23265.86, *p* = 0.002]. *T*-tests performed separately for each SOA condition revealed that for the SOA of 0 ms, responses were faster when sentence direction and effect direction were compatible (*M* = 2148, SD = 220) than when they were incompatible (*M* = 2215, SD = 213), *t*(39) = −2.45, *p* = 0.02. In contrast, for the SOA of 500 ms, responses were slower in trials with compatible directions (*M* = 2178, SD = 217) than in trials with incompatible directions (*M* = 2123, SD = 224), *t*(39) = 2.5, *p* = 0.02. Thus, there was a compatibility advantage when the cue was presented at sentence onset, but when the cue appeared 500 ms after sentence onset, a compatibility disadvantage occurred. In conditions with SOAs of −1000, −500 ms, and 100% of the sentence length, no significant compatibility effects were observed (all |*t*(39)| < 1.8).

#### Error rates

Mean error rates are shown in Table 2. Again, no effect of sentence type was observed; therefore, data are presented averaged over the two sentence types. There was a significant interaction between sentence-effect compatibility and action-effect relation [*F*(1, 38) = 8.04, MSE = 38.89, *p* = 0.007] and a marginally significant interaction between sentence-effect compatibility and SOA [*F*(4, 152) = 2.67, MSE = 50.92, *p* = 0.06]. However, follow-up analyses indicated that the only significant difference between sentence-effect compatibility conditions occurred for the SOA of −1000 ms in the regular condition [*t*(19) = 2.63, *p* = 0.02], even though the interaction between sentence-effect compatibility, action effect relation and SOA did not reach significance [*F*(4, 152) = 0.97]. The error rate was higher in the sentence-effect compatible conditions than in the sentence-effect incompatible conditions. Because significant positive or negative ACEs occurred in different SOA conditions for error rates and for TRTs, a speed-accuracy trade-off can be ruled out.

**Table 2:** **Mean error rates (in %) and standard errors of error rates (in parentheses) in Experiment 2**.

	Compatible	Incompatible
**REGULAR ACTION-EFFECT RELATION**		
SOA = −1000 ms	6.88 (2.37)	1.88 (1.05)
SOA = −500 ms	2.29 (1.30)	0.63 (0.63)
SOA = 0 ms	0.00 (0.00)	0.00 (0.00)
SOA = 500 ms	0.63 (0.63)	0.63 (0.63)
SOA = 100% of the sentence length	0.00 (0.00)	0.00 (0.00)
**INVERTED ACTION-EFFECT RELATION**		
SOA = −1000 ms	3.33 (1.68)	3.33 (1.42)
SOA = −500 ms	1.25 (0.87)	2.50 (1.50)
SOA = 0 ms	0.63 (0.63)	3.75 (1.43)
SOA = 500 ms	0.00 (0.00)	0.83 (0.83)
SOA = 100% of the sentence length	0.00 (0.00)	0.63 (0.63)

*SOA, stimulus onset asynchrony*.

### Discussion

The aim of Experiment 2 was to look in more detail at the temporal dynamics of the interaction between the processes of sentence comprehension and response preparation, in order to investigate which of the conditions might lead to the emergence of a negative ACE. First of all, the results indicate that the timing between sentence comprehension and response preparation does indeed affect whether the ACE is present at all and, if it is present, whether it is positive or negative. When response planning took place 1000 or 500 ms before sentence onset, the ACE was absent in TRTs. When response planning and sentence processing started at the same time, there was a positive ACE in TRTs, whereas the ACE became negative when response planning was delayed for 500 ms. Finally, the ACE disappeared again when response planning took place after the end of the sentence. Because the action-effect relation did not modulate compatibility effects, data indicate that the ACE is related to the intended action effect rather than to the action itself. No significant differences between abstract and concrete sentences were found.

The positive ACE in the condition with the response cue appearing at sentence onset fits well with the TEC-based explanation of the ACE: the cue automatically triggers the activation of the indicated directional feature of the response. We assume that response preparation is completed and feature codes are integrated into the action plan at about 500 ms after the presentation of the cue, because this was the approximate amount of time that passed between the cue presentation at the end of the sentence and the release of the middle button (response initiation). Regarding sentence comprehension, the direction of the described action is clear once subject and verb (the first two words in the sentence) are processed. The end of the verbs lies between 500 ms (for concrete sentences) and 850 ms (for abstract sentences) after sentence onset. Because the uniqueness point at which the verb is recognized and the direction of the described action becomes clear, lies somewhere before this, there seems to be a temporal overlap of the activation of the directional code during response planning and sentence processing. In the sentence-effect compatible condition, priming occurs between the representation of the sentence content and the response representation, because both activate the directional feature code at the same time before it is bound to the one or the other event. Thereby, the comprehension of the sentence is facilitated, which leads to a positive ACE.

The result of the negative ACE that occurred when the response cue was given 500 ms after sentence onset could be explained similarly to Kaschak and Borreggine’s (2008) interpretation of their results: in their experiment, participants were presented with sentences describing transfer toward the body or away from the body, and as a secondary task, compatible or incompatible motor responses had to be executed at different points in time during sentence processing. They found that a positive ACE arose in response times when responses were executed at an early point in the sentences, but disappeared when responses were executed in the middle of the sentences. Similar to the current experiment, responses that were performed 500 ms after the onset of sentences whose length and syntax was comparable to our sentences descriptively displayed sentence-effect compatibility costs. According to the authors, the disappearance of the ACE in the middle of the sentence results from a rather early running of the simulation which might be possible because the last part of the sentences is quite predictable. Thus, in our experiment the activated feature codes may have become integrated into the representation of the sentence content at an early point within the sentence. This might have impaired the preparation of a compatible response^2^.

This explanation also fits well with the finding that the ACE disappeared in the condition in which the cue was presented at the end of the sentence: if the directional feature code needed for planning the response was integrated into the simulation of the sentence content at the end of the sentence, one would have expected interference with response preparation and thus a negative ACE. Yet, if the integration, and with it the temporary binding, of the directional feature code occurred earlier in the sentence, the feature code might become available again for response preparation around the end of the sentence, thereby diminishing the interference effect.

In sum, the results concerning the direction of the ACE are in line with TEC. In addition, the ACE was related to the intended action effect rather than to the action itself. This indicates that representations of intended action effects are activated during the processing of action sentences (regardless of whether they are concrete or abstract), which is also consistent with TEC.

## General Discussion

### Experimental findings

In the present experiments, we were interested in the question of whether the ACE relies on actions or intended action effects. Experiment 1 provided no definite answer to this question but, unexpectedly, showed a negative ACE. Experiment 2 provided evidence that the ACE is related to the intended action effect. Thus, the comprehension of action descriptions involves the activation of action representations referring to the intended effects of these actions. This holds for both concrete and abstract sentences: there were no systematic effects involving sentence type and (particularly in Experiment 2) the ACE was action effect-related for both concrete and abstract sentences.

Experiment 2 additionally addressed the role of timing between sentence comprehension and response preparation in the ACE. We assumed that the negative ACE that occurred in Experiment 1 is caused by preparing the response rather late in the sentence when sufficient information is known to simulate the described action. However, early response preparation was thought to lead to the positive ACE. Consistent with this assumption, the positive ACE emerged when response preparation took place at the beginning of sentence processing, whereas the negative ACE arose when response preparation took place around the middle of the sentence. These findings suggest that the positive ACE is a result of priming between concurrently activated directional feature codes in sentence processing and response planning. In contrast, the negative ACE seems to result from interference between the two processes that probably arises because the directional feature code is already bound to the simulation of the sentence content and, thus, is less accessible for response planning.

### Limitations of the findings

Our data cannot rule out that, in addition to representations of intended action effects, motor representations are also involved in the emergence of the ACE: in some conditions, the ACE was modulated by action-effect relation. However, the modulation by action-effect relation reflected that the ACE occurred only in the inverted action-effect relation condition. It could be that the compatibility effect was more pronounced in this condition because responding was more difficult which resulted in participants concentrating more on the direction of the response. Because of this, the directional feature code might have received stronger activation, which in turn led to stronger interactions with the respective feature code activated during sentence comprehension. However, even though we cannot exclude that motor-related processing of the action sentences did occur in our study, our results show that processing according to intended action effects was stronger. Whether motor programs associated with the described actions are always activated during sentence comprehension and, if so, under which conditions representations of action effects or motor representations dominate, remains an open question.

Overall, the ACEs we observed were weaker and less reliable than the effects found in other ACE experiments (e.g., Glenberg and Kaschak, 2002; Borreggine and Kaschak, 2006; Kaschak and Borreggine, 2008). One reason for this could be that we investigated the ACE in German instead of in English and that differences between the languages could have caused differences in the mental simulation during sentence processing. One of the differences between the English and German linguistic material lies in the sentence construction. In the studies listed above, half of the sentences used the double-object construction (e.g., “Courtney handed you the notebook”/“You handed Courtney the notebook”), and half used the dative construction (e.g., “Andy delivered the pizza to you”/“You delivered the pizza to Andy”), whereas our German sentences were only in the double-object form (e.g., “Andrea bringt dir die Pizza”/“Du bringst Andrea die Pizza”). This was due to the fact that the dative form is not very common in the German language, and for most of the verbs used it would actually be grammatically wrong. Following the linguistic focus hypothesis (Taylor and Zwaan, 2008), this dative form, especially, may give rise to a strong ACE: in this construction, the recipient is postponed to the end of the sentence, whereby the direction of transfer is brought back into the attentional focus at this late point of processing. The renewed activation of the directional feature code around the end of the sentence may enable priming of a compatible response even when response preparation occurs rather late. In contrast, in the German sentences the focus is shifted to the transferred object and not to the direction of transfer at the end of the sentences. This may also contribute to the particular temporal dynamics of the ACE observed in Experiment 2.

### Comparison with related theories

The present findings are compatible with the TEC (Hommel et al., 2001) and, on closer inspection, they are also compatible with the indexical hypothesis by Glenberg and Robertson (1999, 2000). The theory of perceptual symbol systems by Barsalou (1999) appears to be incompatible with the finding that representations of action effects are activated during the comprehension of action sentences.

The correspondence of our results with the common coding approach and with TEC can be explained as follows: extending those theories to linguistic stimuli, we make the additional assumption (following embodied approaches to language comprehension) that semantic meaning of linguistic stimuli is represented in the same format as the perceptual and action events these stimuli refer to. Assuming that the meaning of actions is represented in terms of the intended action effects, it can be claimed that the semantic representations of action words and sentences are shaped by the goals or effects of the described actions. Because of these shared representations of action-related language and real actions, compatibility effects arise between linguistic stimuli and intended action effects. This was confirmed by the appearance of the action effect-related ACE in Experiment 2. The negative ACE and the related time-course of the ACE that we observed in Experiment 2 appear to be broadly consistent with the mechanisms of code activation and integration proposed by TEC.

Similar to the positive ACE, compatibility benefits due to activated codes are also reflected in interactions between language processing and actions that were already mentioned in the introduction (e.g., Boulenger et al., 2006; Zwaan and Taylor, 2006): the representation of the word meaning activates feature codes which then prime feature-overlapping responses. Similar to the negative ACE, compatibility costs due to integrated codes have been shown, for example, between perceptual and action-planning processes (Müsseler and Hommel, 1997): perceptual performance was impaired – the identification of a briefly presented and masked arrow pointing to the left or right – when concurrently preparing a movement that was spatially compatible with the stimulus.

Furthermore, we follow TEC in assuming that the shared representations referring to intended action effects reside on a higher cognitive level. According to Hommel et al. (2001), this is because the activation of representations of intended action effects (distal representations) is assumed to be the initial step in action planning – the more abstract premotor component – and is a prerequisite for selecting the appropriate motor codes (for evidence for shared high-level representations of perception and action see, e.g., Massen and Prinz, 2007). In line with this, some authors propose that more abstract, higher-level action representations might be involved in understanding action-related language (e.g., Zwaan, 1999; de Vega, 2008; Pulvermüller, 2008). According to Zwaan and colleagues (Zwaan, 1999; Taylor and Zwaan, 2009), representations evoked by verbal descriptions can be embodied to different degrees: depending on the existence of one’s own visual or motor experience, the mental representation of the described situation can be rich or poor, detailed or coarse. For example, descriptions of actions that are not part of one’s own action repertoire (such as specific sports) cannot be simulated in detail, and hence the motor system is only slightly involved in their comprehension. Such coarse simulations may draw on higher-level action representations; this might even apply to descriptions of familiar actions when their details remain unspecified (de Vega, 2008).

The finding of the action effect-related ACE contradicts the theory of perceptual symbol systems suggested by Barsalou (1999). According to this theory, linguistic descriptions evoke multimodal representations of their referents, that is, they reactivate associated experiences that are simulated solely by modality-specific systems. In the case of action sentences, mainly experiences of motor states should be simulated. Thus, this approach predicts priming of low-level motor programs (i.e., a movement-related ACE), and it cannot therefore account for the occurrence of the action effect-related ACE.

Similar to Barsalou’s (1999) theory, the indexical hypothesis by Glenberg and Robertson (1999, 2000) also assumes that the words in a sentence activate low-level modal representations of referential objects and actions of the words. Yet, beyond that, the indexical hypothesis proposes two additional processes of sentence comprehension which allow to account for the present results: affordances are derived from these representations, that is, the comprehender gains access to potential motor interactions with the referential objects. As a next step, the affordances are combined or meshed into a coherent, executable, and imaginable set of actions. This meshing process is guided by the meaning of the syntactic construction. For instance, double-object constructions evoke the notion that an agent (Subject) transfers an object or something more abstract (Object2) to a recipient (Object1), that is, they activate a schema for giving (Goldberg, 2003). Thus, syntactic constructions are assumed to activate more generalized action schemas or higher-order action representations (see also Bergen et al., 2004; Feldman and Narayanan, 2004). Correspondingly, the finding of the action effect-related ACE can be explained by the indexical hypothesis in the following way: for one thing, through the meaning of the syntactic construction, processing a transfer sentence activates a certain transfer goal (the action effect). For another thing, affordances are derived depending on current goals and the learning history of the comprehender. If the comprehender has learned that, in the current situation, an action effect in a certain direction can only be achieved by making a movement in the opposite direction, then processing a transfer sentence also activates a movement representation opposite to the direction of transfer. In this way, an action effect-related ACE can occur that relies on both high-level distal representations and low-level motor representations. The indexical hypothesis is therefore consistent with the present results, which point to the importance of high-level representations, as well as with previous results, which point to the importance of motor-level action representations in the comprehension of action-related language (e.g., Glenberg et al., 2008a,b; for a more elaborate account see Glenberg and Gallese, 2012).

## Conclusion

Altogether, our findings confirm the close coupling of cognition and action and provide further evidence for the embodied approach to language comprehension. The presented results revealed that the comprehension of linguistic descriptions of actions involves the activation of higher-order action representations referring to distal effects of these actions.

Moreover, the results indicate that interactions between (declarative) sentence comprehension and (procedural) response selection are highly sensitive to the temporal relationship between the two kinds of processes.

In conclusion, our results suggest that declarative and procedural modes of action processing are less different from each other than is often thought. While they may differ in terms of concomitant mental experiences, they seem to be fairly equivalent in terms of underlying functional mechanisms. They interact with each other and draw on common representational resources, to the effect that high-level processes such as sentence processing can influence action unconsciously. Thus, within the limits of the present paradigm, we can account for our experimental observations without assigning a particular functional (or even causal) role to conscious awareness. However, we do not mean to generalize this conclusion beyond the limits of our paradigm. Other paradigms may invite other kinds of conclusions.

## Conflict of Interest Statement

The authors declare that the research was conducted in the absence of any commercial or financial relationships that could be construed as a potential conflict of interest.

## Footnotes

^1^Preliminary data inspection indicated that participants may not have always selected the response before releasing the middle button (i.e., short response times, RTs), but instead made the decision to press the near or the far button only when they had already initiated the movement. In such instances, response preparation occurs partly during movement time (MT, time from releasing the middle button to pressing the response button) and compatibility effects correspondingly shift from RTs to MTs. We therefore decided to analyze total response time (TRT), the sum of RT and MT.

^2^Although Kaschak and Borreggine define the ACE in terms of movement, it is legitimate to compare our results with theirs, because in their study, as well as in our regular condition, actions and their intended effects were completely correlated and hence sentence-effect compatibility is the same as sentence-action compatibility. Because the ACE did not differ significantly between the regular and the inverted condition in our experiment, the ACE in the inverted condition should also be comparable to Kaschak and Borreggine’s ACE.

## References

[B1] Baddeley A. (2002). “The concept of episodic memory,” in Episodic Memory: New Directions in Research, eds Baddeley A. Conway M. A. Aggleton J. (Oxford: Oxford University Press), 1–10

[B2] Balota D. A. Dolan P. O. Duchek J. M. (2000). “Memory changes in healthy older adults,” in The Oxford Handbook of Memory, eds Tulving E. Craik F. I. M. (New York: Oxford University Press), 395–410

[B3] Barsalou L. W. (1999). Perceptual symbol systems. J. Behav. Brain Sci. 22, 577–660 10.1017/s0140525x99002149 11301525

[B4] Barsalou L. W. (2003). Situated simulation in the human conceptual system. Lang. Cogn. Process. 18, 513–562 10.1080/769813547

[B5] Bergen B. K. Chang N. Narayan S. (2004). “Simulated action in an embodied construction grammar,” in Proceedings of the 26th Annual Conference of the Cognitive Science Society (Mahwah, NJ: Erlbaum), 108–113

[B6] Borreggine K. L. Kaschak M. P. (2006). The action-sentence compatibility effect: it’s all in the timing. Cogn. Sci. 30, 1097–1112 10.1207/s15516709cog0000_91 21702848

[B7] Boulenger V. Roy A. C. Paulignan Y. Deprez V. Jeannerod M. Nazir T. A. (2006). Cross-talk between language processes and overt motor behavior in the first 200 msec of processing. J. Cogn. Neurosci. 18, 1607–1615 10.1162/jocn.2006.18.10.1607 17014366

[B8] Boulenger V. Silber B. Y. Roy A. C. Paulignan Y. Jeannerod M. Nazir T. A. (2008). Subliminal display of action words interferes with motor planning: a combined EEG and kinematic study. J. Physiol. Paris 102, 130–136 10.1016/j.jphysparis.2008.03.015 18485678

[B9] Buccino G. Riggio L. Melli G. Binkofski F. Gallese V. Rizzolatti G. (2005). Listening to action-related sentences modulates the activity of the motor system: a combined TMS and behavioral study. Brain Res. Cogn. Brain Res. 24, 355–363 10.1016/j.cogbrainres.2005.02.020 16099349

[B10] de Vega M. (2008). “Levels of embodied meaning: from pointing to counterfactuals,” in Symbols and Embodiment. Debates on Meaning and Cognition, eds de Vega M. Glenberg A. M. Graesser A. C. (New York: Oxford University Press), 285–308

[B11] Eder A. B. Klauer K. C. (2007). Common valence coding in action and evaluation: affective blindness towards response-compatible stimuli. Cogn. Emot. 21, 1297–1322 10.1080/02699930701438277

[B12] Eder A. B. Klauer K. C. (2009). A common-coding account of the bidirectional evaluation-behavior link. J. Exp. Psychol. Gen. 138, 218–235 10.1037/a0015220 19397381

[B13] Eder A. B. Rothermund K. (2008). When do motor behaviors (mis)match affective stimuli? An evaluative coding view of approach and avoidance reactions. J. Exp. Psychol. Gen. 137, 262–281 10.1037/0096-3445.137.2.262 18473659

[B14] Elsner B. Hommel B. (2001). Effect anticipation and action control. J. Exp. Psychol. Hum. Percept. Perform. 27, 229–240 10.1037/0096-1523.27.1.229 11248937

[B15] Feldman J. Narayanan S. (2004). Embodied meaning in a neural theory of language. Brain Lang. 89, 385–392 10.1016/S0093-934X(03)00355-9 15068922

[B16] Glenberg A. M. Gallese V. (2012). Action-based language: a theory of language acquisition, comprehension, and production. Cortex 48, 905–922 10.1016/j.cortex.2011.04.010 21601842

[B17] Glenberg A. M. Kaschak M. P. (2002). Grounding language in action. Psychon. Bull. Rev. 9, 558–565 10.3758/BF03196313 12412897

[B18] Glenberg A. M. Robertson D. A. (1999). Indexical understanding of instructions. Discourse Process. 28, 1–26 10.1080/01638539909545067

[B19] Glenberg A. M. Robertson D. A. (2000). Symbol grounding and meaning: a comparison of high-dimensional and embodied theories of meaning. J. Mem. Lang. 43, 379–401 10.1006/jmla.2000.2714

[B20] Glenberg A. M. Sato M. Cattaneo L. (2008a). Use-induced motor plasticity affects the processing of abstract and concrete language. Curr. Biol. 18, 290–291 10.1016/j.cub.2008.02.036 18397734

[B21] Glenberg A. M. Sato M. Cattaneo L. Riggio L. Palumbo D. Buccino G. (2008b). Processing abstract language modulates motor system activity. Q. J. Exp. Psychol. 61, 905–919 10.1080/17470210701625550 18470821

[B22] Goldberg A. E. (2003). Constructions: a new theoretical approach to language. Trends Cogn. Sci. (Regul. Ed.) 7, 219–224 10.1016/S1364-6613(03)00080-9 12757824

[B23] Hommel B. (1993). Inverting the Simon effect by intention. Determinants of direction and extent of effects of irrelevant spatial information. Psychol. Res. 55, 270–279 10.1007/BF00419608

[B24] Hommel B. Müsseler J. Aschersleben G. Prinz W. (2001). The theory of event coding (TEC): a framework for perception and action planning. J. Behav. Brain Sci. 24, 849–937 10.1017/S0140525X01000103 12239891

[B25] Kaschak M. P. Borreggine K. L. (2008). Temporal dynamics of the action-sentence compatibility effect. Q. J. Exp. Psychol. 61, 883–895 10.1080/17470210701623852 PMC4612619 18470819

[B26] Lindemann O. Stenneken P. van Schie H. T. Bekkering H. (2006). Semantic activation in action planning. J. Exp. Psychol. Hum. Percept. Perform. 32, 633–643 10.1037/0096-1523.32.3.633 16822129

[B27] Markman A. B. Brendl C. M. (2005). Constraining theories of embodied cognition. Psychol. Sci. 16, 6–10 10.1111/j.1467-9280.2005.01625.x 15660844

[B28] Massen C. Prinz W. (2007). Activation of action rules in action observation. J. Exp. Psychol. Learn. Mem. Cogn. 33, 1118–1130 10.1037/0278-7393.33.6.1118 17983317

[B29] Müsseler J. Hommel B. (1997). Blindness to response-compatible stimuli. J. Exp. Psychol. Hum. Percept. Perform. 23, 861–872 10.1037/0096-1523.23.3.861 9180047

[B30] Prinz W. (1990). “A common coding approach to perception and action,” in Relationships Between Perception and Action. Current Approaches, eds Neumann O. Prinz W. (Berlin: Springer), 167–201

[B31] Prinz W. (1997). Perception and action planning. Eur. J. Cogn. Psychol. 9, 129–154 10.1080/713752551

[B32] Pulvermüller F. (2005). Brain mechanisms linking language and action. Nat. Rev. Neurosci. 6, 576–582 10.1038/nrn1706 15959465

[B33] Pulvermüller F. (2008). “Grounding language in the brain,” in Symbols and Embodiment. Debates on Meaning and Cognition, eds de Vega M. Glenberg A. M. Graesser A. C. (New York: Oxford University Press), 85–116

[B34] Pulvermüller F. Hauk O. Nikulin V. V. Ilmoniemi R. J. (2005). Functional links between motor and language systems. Eur. J. Neurosci. 21, 793–797 10.1111/j.1460-9568.2005.03900.x 15733097

[B35] Richardson D. C. Spivey M. J. Cheung J. (2001). “Motor representations in memory and mental models: embodiment in cognition,” in Proceedings of the Twenty-third Annual Conference of the Cognitive Science Society, eds Moore J. D. Stenning K. (Mahwah, NJ: Lawrence Erlbaum Associates), 867–872

[B36] Rieger M. (2007). Letters as visual action effects in skilled typing. Acta Psychol. (Amst) 126, 138–153 10.1016/j.actpsy.2006.11.006 17250793

[B37] Sereno S. C. Rayner K. Posner M. I. (1998). Establishing a time-line of word recognition: evidence from eye movements and event-related potentials. Neuroreport 9, 2195–2200 10.1097/00001756-199807130-00009 9694199

[B38] Squire L. R. (1992). Memory and the hippocampus: a synthesis from findings with rats, monkeys, and humans. Psychol. Rev. 99, 195–231 10.1037/0033-295X.99.3.582 1594723

[B39] Stoet G. Hommel B. (2002). “Interaction between feature binding in perception and action,” in Common Mechanisms in Perception and Action: Attention and Performance XIX, eds Prinz W. Hommel B. (Oxford: Oxford University Press), 538–552

[B40] Taylor L. J. Zwaan R. A. (2008). Motor resonance and linguistic focus. Q. J. Exp. Psychol. 61, 896–904 10.1080/17470210701625519 18470820

[B41] Taylor L. J. Zwaan R. A. (2009). Action in cognition: the case of language. Lang. Cogn. 1, 45–58

[B42] Tettamanti M. Buccino G. Saccuman M. C. Gallese V. Danna M. Scifo P. (2005). Listening to action-related sentences activates fronto-parietal motor circuits. J. Cogn. Neurosci. 17, 273–281 10.1162/0898929053124965 15811239

[B43] Tulving E. (2000). “Concepts of memory,” in The Oxford Handbook of Memory, eds Tulving E. Craik F. I. M. (New York: Oxford University Press), 33–44

[B44] van Dantzig S. Pecher D. Zwaan R. A. (2008). Approach and avoidance as action effects. Q. J. Exp. Psychol. 61, 1298–1306 10.1080/17470210802027987 19086189

[B45] Zwaan R. A. (1999). Embodied cognition, perceptual symbols, and situation models. Discourse Process. 28, 81–88 10.1080/01638539909545070

[B46] Zwaan R. A. (2004). “The immersed experiencer: toward an embodied theory of language comprehension,” in The Psychology of Learning and Motivation: Advances in Research and Theory, Vol. 44, ed. Ross B. H. (New York, NY: Elsevier Science), 35–62

[B47] Zwaan R. A. Taylor L. J. (2006). Seeing, acting, understanding: motor resonance in language comprehension. J. Exp. Psychol. Gen. 135, 1–11 10.1037/0096-3445.135.1.1 16478313

[B48] Zwaan R. A. van der Stoep N. Guadalupe T. Bouwmeester S. (2012). Language comprehension in the balance: the robustness of the action-compatibility effect (ACE). PLoS ONE 7:e31204 10.1371/journal.pone.0031204 22363580 PMC3283597

